# Clival Chondroid Chordoma: A Case Report and Review of the Literature

**DOI:** 10.7759/cureus.3381

**Published:** 2018-09-28

**Authors:** Ibeth S Erazo, Claudio F Galvis, Luis E Aguirre, Roman Iglesias, Luz C Abarca

**Affiliations:** 1 Department of Emergency Medicine, Hospital Teodoro Maldonado Carbo / Universidad Catolica De Santiago De Guayaquil, Guayaquil, ECU; 2 Department of Internal Medicine, University of Miami Miller School of Medicine/Jackson Memorial Hospital, Miami, USA; 3 Department of Pathology, Hospital Teodoro Maldonado Carbo / Universidad Catolica De Santiago De Guayaquil, Guayaquil, ECU; 4 Department of Internal Medicine, Hospital Teodoro Maldonado Carbo / Universidad Catolica De Santiago De Guayaquil, Guayaquil, ECU

**Keywords:** spine chordoma, chondroid chordoma, bone tumor, adolescent pediatrics, rare cases, neurological injury

## Abstract

Chordomas are rare, slow-growing, and locally aggressive malignant neoplasms derived from primitive notochord remnants. The chondroid variety represents 14% of all chordomas mainly developing in the spheno-occipital region and presenting between the third and fifth decades of life. When developing intracranially, symptoms can range from headaches and neck pain to cranial nerve neuropathies and facial numbness. We illustrate a case of an adolescent woman who presented with excruciating facial pain, otalgia, decreased visual acuity, quadriparesis, headache, nausea, and dysphagia. Radiological studies revealed a large heterogeneous mass in the spheno-occipital region with clivus destruction. Biopsy and histopathology confirmed the diagnosis. Proper identification with prompt surgical resection and adjuvant radiotherapy remains critical to prevent complications.

## Introduction

Chordomas are rare, slow-growing, and locally aggressive malignant neoplasms derived from primitive notochord remnants that develop longitudinally along the axial axis. They represent 1%-4% of all primary bone tumors, with 35% of cases presenting at the base of the skull. The chondroid variety represents 14% of all chordomas and is generally found in patients between the third and fifth decades of life [1-3]. They mainly appear in the spheno-occipital region, representing 35% of all local chordomas. Clinical presentation of these tumors is highly dependent on anatomical location [1]. These neoplasms tend to be less aggressive than their conventional counterparts and are associated with a better prognosis [1, 4]. They can be further classified into classic, chondroid, and undifferentiated subtypes. Imaging studies such as magnetic resonance imaging (MRI) and computed tomography (CT) of the skull are essential for proper diagnosis and surgical planning [2, 5]. Biopsy and immunohistochemistry are critical for diagnosis. Chordomas are resistant to chemotherapy, and thus, the treatment of choice lies in complete surgical resection usually followed by adjuvant radiotherapy [6-8]. Surgery for clival chordoma is challenging because infiltration to the surrounding neurovascular structures is frequent, and deterioration of the quality of life may be considerable.

## Case presentation

**Figure 1:**
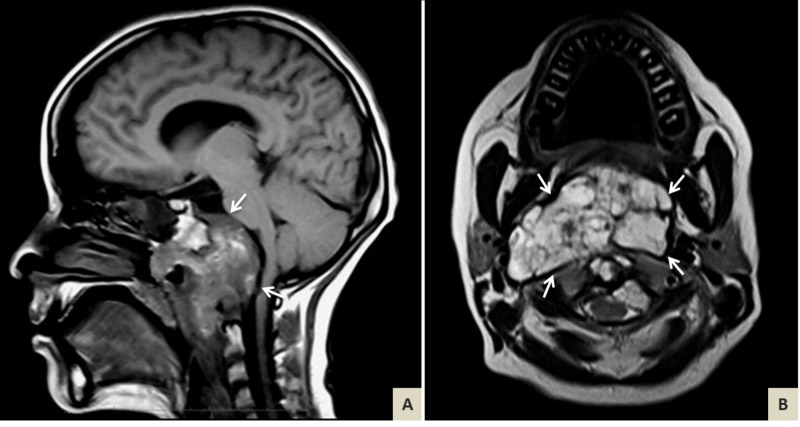
Brain magnetic resonance imaging (MRI) prior to surgery. (A) T1-weighted sagittal and (B) T2-weighted axial MRI showing heterogeneous enhancement with honeycomb appearance of a poorly defined spheno-occipital mass invading and eroding the clivus (white arrows). Features are consistent with areas of tumor hemorrhage and calcification. The pons and medulla are displaced.

A 15-year-old female with no past medical history presented to the emergency department complaining of five months of severe pain in nasal region and associated cutaneous hypersensitivity, headache, nausea, dysphagia, otalgia, strabismus of left eye with decreased visual acuity, and quadriparesis. On physical exam, she presented with horizontal nystagmus, dysarthria, quadriparesis, and a palpable mass on the right side of the neck with neck stiffness. An MRI of the brain and neck revealed an enhanced broad and destructive mass in the infrasellar region with complete destruction of the clivus, C1-C2 infiltration with compression of the occipital foramen, and a displaced pons and medulla (Figure 1).

**Figure 2:**
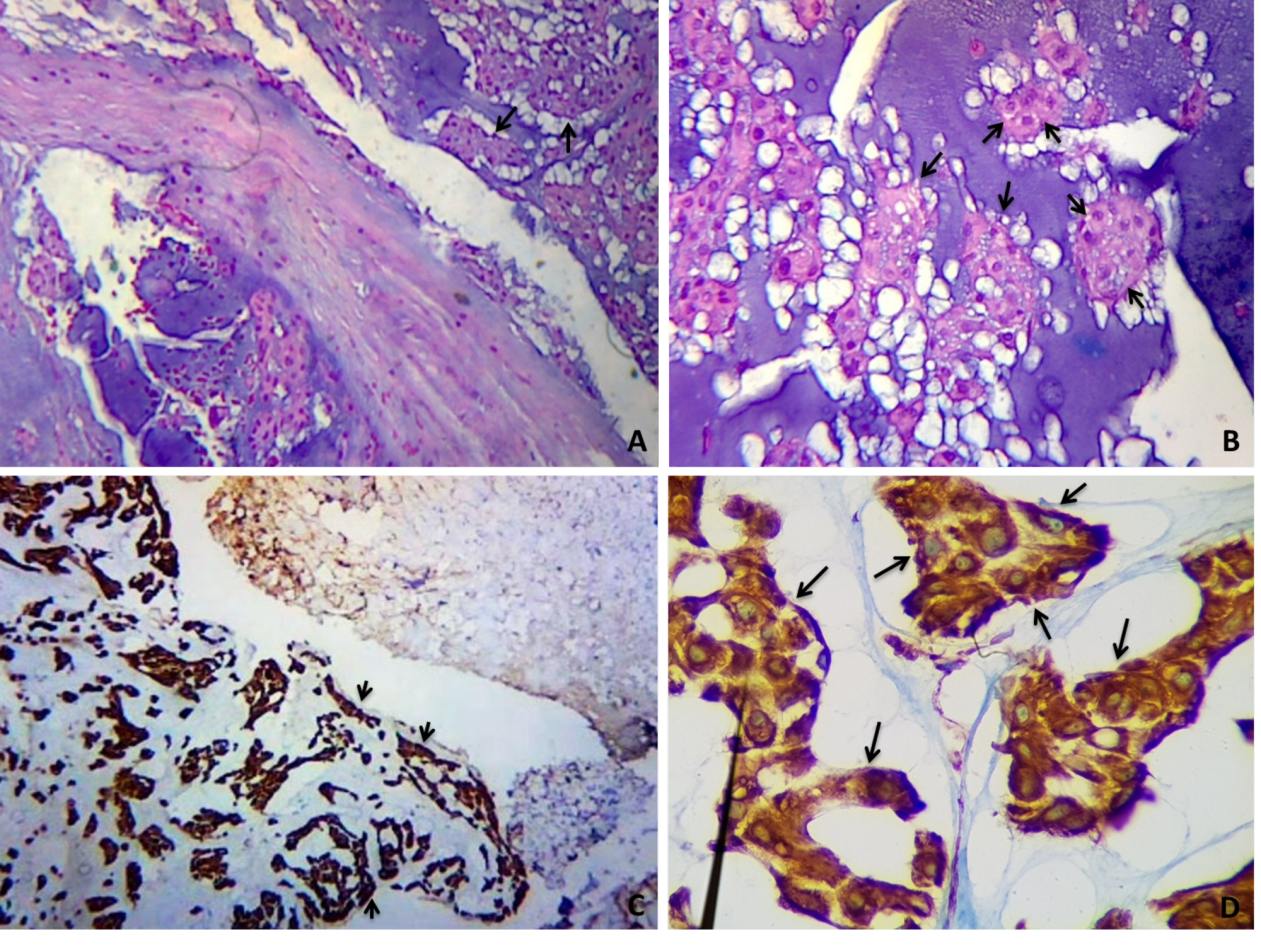
Chondroid chordoma (pathology slides). (A, B) Hematoxylin and eosin (HE) stains at 40x and 100x magnification reveal nests of cells with large, pleomorphic nuclei and partly vacuolated cytoplasm rich in mucopolysaccharides (physalipherous cells). Photomicrographs show typical chordoma cells forming sheets or lobular structures embedded in a mucoid stroma (black arrows). (C, D) Cytokeratine AE1/AE3 stain at 40x and 400x magnification. Antibodies against keratin show the ratio of neoplastic cells (dark brown) and interstitial tissue (in blue). Lobules of tumor tissue appear surrounded by strands of fibrous tissue (black arrows).

The patient underwent a two-stage procedure. The first procedure was a transoral approach with decompressive partial excision surgery. A sample biopsy of the lesion was taken, which was found to be consistent with chondroid chordoma. Further biopsies were obtained and the pathology studies resulted positive for S100, cytokeratin AE1/AE3, and epithelial membrane antigen (EMA). Microscopically polygonal cells with hyperchromatic nuclei, fine cytoplasmic vacuoles, and chondroid tissue corresponding to phisaliferous cells constituting nests and lobules infiltrated and replaced the hyaline bone cartilage and part of the soft tissue (Figure 2).

**Figure 3:**
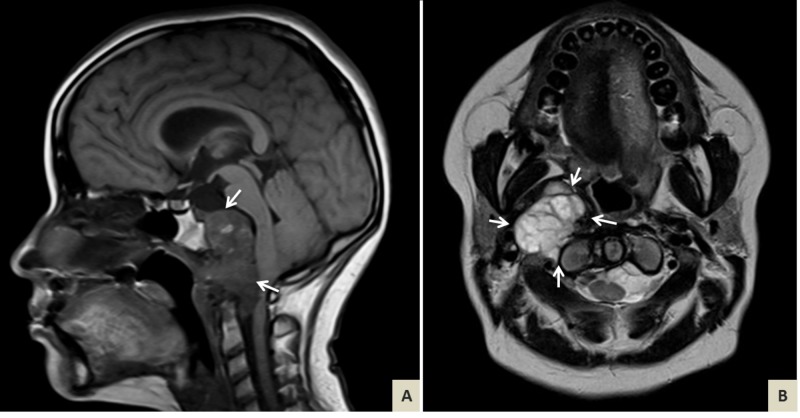
Brain magnetic resonance imaging (MRI) postsurgery. (A) T1-weighted sagittal and (B) T2-weighted axial MRI after partial excision show decompression of pons and medulla following reduction of tumor burden (white arrows).

The second procedure was performed one month later and consisted of maxillectomy Le-fort 1. Partial excision of residual tumor mass was successfully achieved. After three days of postoperative recovery, the patient showed signs of partial improvement. She was discharged to follow up with imaging monthly (Figure 3).

## Discussion

Chordomas are rare malignant neoplasms that develop from the primitive notochord and present as local, slowly infiltrative lesions. Chordomas can occur at any age but are usually seen in Caucasian adults between the ages of thirty and seventy years. Those located in the sacrococcygeal region constitute 50% of all cases and are typically seen in a slightly older age group with highest frequency within the fifth decade of life [9]. In contrast, 35% of cases occur at the base of the skull, most commonly among patients 20-40 years of age. Furthermore, 15% of cases originate in the spine [10-12]. 

The most common locations involve the head and neck, specifically the clivus, spinal-occipital synchondrosis of the clivus, and sella turcica. Per histopathology and immunohistochemistry techniques, they can be further classified into classic, chondroid, and undifferentiated chordomas [3, 5, 13]. Clinical manifestations depend on the area of localization of the tumor mass. Intracranial chordomas can present with headaches, neck pain, cranial nerve neuropathies, diplopia, facial numbness, and even hypopituitarism if the sella turcica is involved [1]. 

Imaging studies such as MRI and CT of the skull are essential for proper diagnosis and surgical planning. Biopsy and immunohistochemistry contribute to final diagnosis.

Chordomas appear as destructive lytic lesions at the base of the skull and soft tissue masses with occasional dystrophic calcifications [8-9]. MRI shows hypointensity of the mass in T1-weighted and hyperintensity in T2-weighted sequences. MRI is preferred over CT imaging as it allows better delimitation of the tumor mass and assessment of its relationship to the adjacent vasculature [1, 9]. Macroscopically, they are gray-blue lobulated tumors with a soft consistency. Histologically, tissue shows abundant production of the intracellular mucinous matrix, phisalifera cells, and cartilaginous foci. Chordomas are characterized by lobulated arrangements of cells with a tendency to form bands or pseudoacinar structures [6, 9]. Immunohistochemistry is positive for cytokeratin and EMA, S100, and vimetine [1-2, 13]. 

While chordomas are resistant to chemotherapy, the treatment of choice lies in complete surgical resection in blocks usually followed by adjuvant radiotherapy [6-8]. Of the latter, multiple modalities have been described ranging from proton-beam radiotherapy to newer ones such as fractionated photon radiotherapy, carbon-ion radiotherapy, and stereotactic radiosurgery [10]. Proton-beam therapy delivers high fractions of radiation to target tissues while minimizing toxic exposure to local peritumoral structures [10]. Carbon-ion therapy exerts a similar effect while exhibiting higher relative biological effectiveness [10]. Stereotactic radiosurgery, on the other hand, remains an attractive option for smaller tumors or when addressing recurrent disease [10]. To date no superiority of any treatment modality has been demonstrated over the other [10]. Furthermore, there is lack of proper consensus with regard to defining an optimal dosing protocol and evidence remains limited [10].

Treatment of intracranial chordomas can be restricted by their common location near sensitive structures such as the brain stem, optic nerve, optic chiasm, cochlea, pituitary gland, and temporal lobes [8, 10, 14, 15]. The main challenge of administering safe and effective radiotherapy is to achieve clinically relevant doses while mitigating the damage to adjacent neurovascular structures, particularly the optic nerve, pituitary gland, and brainstem [1, 6]. 

The probability of recurrence remains elevated even after total resection, with recurrence rate reported as high as 68% and associated with a poor prognosis. The natural history of chordomas remains unpredictable. The rate of distant metastasis is reported between 5% and 29%. Five-year survival rates vary from 51% to 82% according to histological grade and absence of positive surgical margins [2]. 

## Conclusions

Chordoma cases remain extremely rare in adolescents, as they tend to occur after the third decade of life. These tumors mostly manifest sporadically, hence posing a clinical challenge to general practitioners. Our main goal was to briefly review primary characteristics of chondroid chordomas, diagnostic tools needed, and management. Imaging studies, including MRI of the brain are essential for surgical planning, assessment of growth pattern, and integrity of critical anatomical landmarks. Chondroid chordomas usually have a better prognosis when compared to other types. Surgical resection followed by postoperative radiotherapy remains the treatment of choice.
